# Germinal Center B Cell and T Follicular Helper Cell Responses to Viral Vector and Protein-in-Adjuvant Vaccines

**DOI:** 10.4049/jimmunol.1502472

**Published:** 2016-07-13

**Authors:** Chuan Wang, Matthew Hart, Cecilia Chui, Augustine Ajuogu, Iona J. Brian, Simone C. de Cassan, Persephone Borrow, Simon J. Draper, Alexander D. Douglas

**Affiliations:** * Jenner Institute, Nuffield Department of Medicine, University of Oxford, Oxford OX3 7DQ, United Kingdom; and; † Nuffield Department of Medicine, University of Oxford, Oxford OX3 7FZ, United Kingdom

## Abstract

There is great interest in the development of Ab-inducing subunit vaccines targeting infections, including HIV, malaria, and Ebola. We previously reported that adenovirus vectored vaccines are potent in priming Ab responses, but uncertainty remains regarding the optimal approach for induction of humoral immune responses. In this study, using OVA as a model Ag, we assessed the magnitude of the primary and anamnestic Ag–specific IgG responses of mice to four clinically relevant vaccine formulations: replication-deficient adenovirus; modified vaccinia Ankara (a poxvirus); protein with alum; and protein in the squalene oil-in-water adjuvant Addavax. We then used flow cytometric assays capable of measuring total and Ag-specific germinal center (GC) B cell and follicular Th cell responses to compare the induction of these responses by the different formulations. We report that adenovirus vectored vaccines induce Ag insert–specific GC B cell and Ab responses of a magnitude comparable to those induced by a potent protein/squalene oil-in-water formulation whereas—despite a robust overall GC response—the insert-specific GC B cell and Ab responses induced by modified vaccinia Ankara were extremely weak. Ag-specific follicular Th cell responses to adenovirus vectored vaccines exceeded those induced by other platforms at day 7 after immunization. We found little evidence that innate immune activation by adenovirus may act as an adjuvant in such a manner that the humoral response to a recombinant protein may be enhanced by coadministering with an adenovirus lacking a transgene of interest. Overall, these studies provide further support for the use of replication-deficient adenoviruses to induce humoral responses.

## Introduction

For many infections—notably malaria and HIV but also numerous further diseases of humans and livestock—the induction of Ab responses by recombinant subunit vaccines is the leading approach to the development of an efficacious vaccine. Advantages of subunit vaccine approaches over live-attenuated and killed vaccines include the ability to focus immune responses upon a tailor-made immunogen, for example, designed to elicit responses to neutralizing or conserved epitopes. There is therefore intense interest in the development of subunit vaccine approaches with optimal humoral immunogenicity, with areas of particular interest including the optimization of peak Ab titers, recall responses to Ag, somatic hypermutation, and long-term maintenance of Ab responses.

To meet these requirements, numerous vaccine delivery platforms are under investigation. A considerable array of immunostimulating adjuvant approaches suitable for formulation with recombinant protein Ags have reached various stages of clinical and preclinical development (1, 2). In parallel, extensive efforts have been made to develop replication-deficient viral vector vaccine platforms that are capable of delivering an Ag encoded as a transgene. Although originally developed primarily for their capacity to induce strong cellular immune responses—particularly CTL responses—there has more recently been considerable interest in the capacity of some viral vector vaccines to induce potent humoral responses (3–6).

Previous data from studies conducted by our group in mice, rhesus macaques, and humans have suggested particular advantages of regimes in which a replication-deficient adenovirus is used as a priming vaccine followed by a boost vaccine delivering the same Ag in a different manner (either protein/adjuvant or a heterologous viral vector such as modified vaccinia Ankara [MVA]) (7–11). In such regimes, the use of the adenoviral prime appeared to overcome the need to formulate the boosting immunogen with a potent adjuvant to reach very high postboost Ab titers; in other words, regimes using an adenovirus prime followed by a boost using recombinant protein in an adjuvant conventionally regarded as relatively weak were capable of inducing Ab titers which matched those induced by the most potent protein/adjuvant regimes (8, 11). These studies did not address the mechanism by which this effect was achieved.

Although there has been detailed study of the process by which viral vector vaccines induce T cell responses, there has been relatively little exploration of the process by which these vaccines induce humoral responses. Dramatically different transgene (Ag) expression kinetics have been demonstrated after immunization with replication-deficient adenovirus and poxvirus vectors, with the former achieving high levels of Ag expression for >10 d, whereas MVA induces a brief high-level burst of Ag expression that appears beneficial for CTL induction but may not achieve sufficient levels of free Ag for optimal humoral responses (12, 13). Elegant studies have delineated a number of pathways of innate immune activation that contribute to the immunogenicity of adenovirus vectors, with roles for TLR9-mediated plasmacytoid dendritic cell activation, TLR2-driven NF-κB activation, and TLR-independent activation of type I IFN driving signaling to both B and CD4^+^ T cells (14–17).

The germinal center (GC), in which Ag-specific B cells receive help from cognate CD4^+^ T follicular helper (Tfh) cells, is central to the humoral immune response, the principal site of somatic hypermutation, and the main source of memory B cells and long-lived plasma cells in the response to T-dependent protein Ags (18, 19). There is, to our knowledge, little data comparing viral vector vaccines versus protein/adjuvant vaccines for their ability to induce GC B cell and Tfh responses (20). Such comparisons are hindered by the fact that, in contrast to protein-only immunization, a proportion of the total immune response to viral vector vaccination is directed against structural components of the vector rather than toward the desired target (the Ag expressed from the inserted transgene). In this study, we made use of flow cytometric assays designed to identify Ag-specific GC B cells and Tfh cells to compare the induction of these cell populations by a variety of viral vector and protein/adjuvant vaccine platforms.

We further extended these studies to investigate the possibility that, given the known ability of adenovirus to activate innate immunity, a non-antigen encoding adenovirus could act as an adjuvant when coadministered with soluble protein: in other words, the Ag and the adenovirus need not be administered in *cis* but could be administered in *trans*. We show that, in fact, there is little or no such *trans* adjuvanting effect on Ab, GC, or Tfh induction; it appears that the ability of adenovirus to induce robust humoral responses is dependent on expression of the Ag within the same cells infected by the adenovirus.

## Materials and Methods

### Vaccine selection

As stated in *Results*, a comprehensive comparison of viral vector vaccines with protein-in-adjuvant formulations is clearly impossible. Selection of appropriate vectors, adjuvants, and doses is required and is complicated by difficulty in extrapolating from doses used in mice to those suitable for use in humans. The following section sets out our rationale for the choice of the vaccine platforms compared in this study, and the doses used.

Humoral responses to vaccination generally rise with increasing Ag dose, with little evidence that higher doses are deleterious. For each of the platforms we wished to study, we therefore selected doses at the upper end of the range of doses suitable for use in mice to obtain optimal responses.

Adenovirus is probably the leading viral vector platform for Ab response induction, having reached clinical trials for a variety of major diseases and attracted the interest of leading pharmaceutical companies. To represent this approach, we used human adenovirus serotype 5 (AdHu5) expressing the model Ag OVA. AdHu5 is the most widely studied adenovirus vaccine vector, and although its use in humans is limited by the prevalence of anti-AdHu5 neutralizing Abs, it is among the most immunogenic adenovectors in mice and primates (21–23). There are a variety of differences between and within adenovirus species: as a member of adenovirus species C, AdHu5 is similar both at the sequence level and in terms of immunogenicity to clinically used adenovirus serotypes such as ChAd3, PanAd3, and human adenovirus serotype 6 (21). Throughout, we used a dose of 1 × 10^8^ infectious units for human adenovirus serotype 5 expressing OVA (AdHu5-OVA); for the virus preparation used, this corresponded to 4.3 × 10^9^ virus particles. This is at the upper end of the range of adenovirus doses used in mouse studies by our group or other investigators (8, 24), ∼2% of the highest adenovirus-vectored vaccine dose we are aware has been used in humans, and 5–10% of a typical human dose (10, 25, 26).

Poxvirus vectors have also been employed as vaccine vectors with the aim of Ab induction, including in clinical trials and in a vaccinia-based licensed veterinary vaccine against rabies (6, 27). For this study, we selected MVA to represent the group of replication-deficient poxviruses in development for human vaccines: MVA is among the most studied poxvirus vectors, with transgene–Ag immunogenicity favorably comparable with other poxviruses (5, 6, 28). MVA was dosed at 1 × 10^7^ PFU, also at the upper end of the range of doses used in previously published mouse studies (7, 8), ∼2% of the highest dose we are aware of being used in humans, and 5–10% of a typical human dose (10, 26, 29). Although there are a limited number of examples of MVA doses > 1 × 10^7^ PFU being used in mice, published data suggest that 1 × 10^7^ PFU is sufficient to achieve optimal humoral responses when delivered by the i.m. route (as opposed to the suboptimal s.c. or clinically less relevant i.p. route) (30, 31). The MVA used here expressed GFP as a marker; we have previously produced both GFP-expressing and markerless versions of several MVA viruses and observed no detectable difference in Ag transgene immunogenicity (32) (and further data not shown).

Protein doses used for mouse immunization in the literature vary by several orders of magnitude; in this study, we selected a dose of 10 μg of OVA (InvivoGen), again toward the upper end of the typically used range (7, 8, 33). As an adjuvant for protein vaccines, we first selected alum, which is the most widely used adjuvant in current licensed vaccines. Al(OH)_3_-based alum adjuvant (Alhydrogel; InvivoGen) was chosen in preference to aluminum phosphate because of previous data demonstrating superior immunogenicity of the former (presumably because of adsorption of the negatively charged OVA protein to the positively charged Al(OH)_3_); 85 μg of Al^3+^ was given per dose, again consistent with our previous work (8). As a second clinically plausible adjuvant, we selected a squalene oil-in-water (o/w) emulsion (Addavax; InvivoGen), dosed according to the manufacturer’s recommendation. Proprietary but similar squalene o/w formulations, such as MF59 (Novartis) and AS03 (GSK), are among the few relatively novel adjuvant formulations, which are currently used in licensed vaccines (34, 35).

### Western blotting

To demonstrate Ag expression by the AdHu5-OVA and MVA-OVA vectors, we detected OVA expression by Western blotting of the supernatants of infected cell cultures. HEK293A cells were cultured in RPMI 1640 medium with 10% FCS and infected with either AdHu5-OVA or AdHu5 expressing the irrelevant malaria Ag PfAMA1 (36) at a multiplicity of infection of 10 infectious units/cell. BHK cells were cultured similarly and infected with either MVA-OVA or a MVA-PfAMA1 (36) at a multiplicity of infection of 10 PFU/cell. Cell supernatant and infected cells were collected separately 8 and 24 h postinfection, boiled with XT reducing agent and Laemlli buffer (both from Bio-Rad), run on a Novex NuPAGE Bis-Tris SDS 4–12% gel (ThermoFisher), transferred to nitrocellulose membrane using a TransBlot Turbo system (Bio-Rad), and blotted using an iBind system (ThermoFisher). Serum from mice immunized with OVA protein was used as primary Ab, with detection using alkaline-phosphatase–conjugated donkey-anti-mouse IgG (The Jackson Laboratory) and BCIP/NBT (Sigma-Aldrich). Recombinant OVA (InvivoGen) was used as a positive control.

### Animals and immunizations

All animal work was approved by the University of Oxford Animal Welfare and Ethical Review Body (in its review of the application for the U.K. Home Office Project License PPL 30/2889). Female C57BL/6 mice (Harlan) were housed in a specific-pathogen-free facility and were 6–8 wk old at the initiation of each experiment.

The production of the AdHu5-OVA, AdHu5-empty, and MVA-OVA vaccines has been reported elsewhere (8, 37). In brief, the OVA transgene included the human tissue plasminogen activator signal sequence and the full-length chicken OVA sequence, with an N311D amino acid substitution. This substitution has previously been reported to prevent N-linked glycosylation at this residue, resulting in expression from mammalian cells of protein with a pattern of glycosylation matching that of hen egg OVA (38). In the AdHu5-OVA vector, the transgene was driven by an intron-containing CMV immediate-early promoter; this promoter (or closely related variants) has been used in the majority of adenoviruses, which have entered clinical trials (39). The empty AdHu5 included the transgene promoter and polyA tail but no Ag-coding sequence. Adenoviral vaccines were grown in HEK293 T-REx cells (Life Technologies), purified by CsCl centrifugation, and titered both by UV spectrophotometry to measure viral particles (per milliliter) and by hexon immunostaining to assess infectious units (per milliliter). Particle:infectious unit ratios were 43 for AdHu5-OVA and 36 for AdHu5-empty. In the MVA-OVA vector, the transgene was driven by the p7.5 promoter inserted at the thymidine kinase locus; this is the most widely used promoter for recombinant poxvirus vaccine vectors (40, 41). MVA vaccines were grown in chicken embryo fibroblasts, purified by centrifugation through a sucrose cushion and titered by fluorescence plaque assay using the GFP marker to measure PFUs (per milliliter). Endotoxin-free chicken OVA and adjuvants were purchased from InvivoGen (Endofit OVA, Addavax and Alhydrogel, which is a colloidal suspension of aluminum hydroxide). Doses used were 1 × 10^8^ infectious units for AdHu5, 1 × 10^7^ PFU for MVA, 10 μg of OVA protein, 25 μl of Addavax, and 85 μg of Al^3+^ in the form of Al(OH)_3_; the rationale for selection of these doses is explained above. Protein–adjuvant formulations were prepared in accordance with the manufacturer’s recommendations. All vaccinations were diluted in PBS to a total volume of 50 μl, which was administered i.m., split equally between the gastrocnemius muscles of each hind limb.

### ELISA

ELISA was performed essentially as described previously (42, 43). In brief, plates were coated with OVA protein (InvivoGen Endofit, 50 μl/well at 1 μg/ml). Dilutions of test sera (in triplicate) and a standard curve produced using serial dilutions of a reference serum pool from OVA–immune mice were added to the plate. Washing, secondary Ab binding, final washing, and detection were all as previously described, with the exception that the secondary Ab used was alkaline-phosphatase–conjugated goat anti-mouse IgG (Sigma-Aldrich). OD_405_ was quantified using an ELx800 plate reader (Bio-Tek). Results were expressed in Ab units (AU), defined using the reference standard, by interpolation of OD_405_ readings on the standard curve.

### Immunohistology

Inguinal lymph nodes were harvested, immersed in Optimum Cutting Temperature (OCT) compound (VWR Chemicals), and immediately frozen on dry ice. Thirty-five-micrometer sections were cut using a cryostat (Leica), mounted on Superfrost Ultra Plus slides (Thermo Scientific Gerhard Menzel), fixed in acetone for 10 min at −20°C, air-dried for 30 min, and stored at −20°C.

Prior to staining, non-specific Ab binding was blocked using a solution of 1% v/v normal mouse serum, 2% w/v BSA (Sigma-Aldrich), and 0.3% v/v Triton X-100 (Sigma-Aldrich). Primary Abs and secondary reagent were used as detailed in Supplemental Table I. After final washing, Fluoromount G mounting medium (eBioscience) was applied, followed by a coverslip.

Slides were visualized using a Zeiss LSM 710 confocal microscope. Images were acquired using Zen software (Zeiss). ImageJ (National Institutes of Health) was used for image processing. Images were analyzed in a manner allowing comparison of both overall GC area and proportion of the B cell area occupied by GCs: GC and B cell areas were identified using GL7 and B220 staining, respectively, manually defined, and their areas were calculated (44).

### Flow cytometry

Popliteal, inguinal, and para-aortic lymph nodes were all noted to be enlarged in response to immunization and were therefore together considered to be relevant draining lymph nodes (DLNs), which were harvested and stored on ice in RPMI 1640 medium for no more than 3 h before preparation of single-cell suspensions.

Staining reagents were used as set out in Supplemental Table I. GL7 was chosen as a GC B cell marker.

MHC class II (MHC-II) tetramers (each comprising a peptide displayed on H-2 I-A^b^ and tetramerized with PE-labeled streptavidin [SA-PE]) were obtained from the NIH Tetramer Core Facility (Emory University). For staining of OVA-reactive CD4^+^ T cells, a mixture was prepared comprising tetramers displaying the peptides TEWTSSNVMEERKIKV (OVA residues 266–281), AAHAEINEA (OVA residues 329–337), and HAAHAEINEA (OVA residues 328–337).

To prepare B cell tetramers, HEK293E cells were transiently cotransfected with plasmids encoding the BirA biotin ligase and OVA with C-terminal biotin acceptor peptide and C-tag (peptide EPEA). Following expression, purification of protein was performed using CaptureSelect Ctag resin (Life Technologies). Purified protein was extensively dialyzed and incubated with SA-PE (Life Technologies). Residual unreacted SA-PE and OVA were removed by size exclusion chromatography using a Superdex 200 column (GE). Prior to use, the reagent was confirmed to stain beads coated with anti-OVA mouse serum.

In the case of B cell staining, a single-step surface and viability stain was carried out in PBS, preceded by blocking of Fc-mediated Ab binding with anti-CD16/ anti-CD32 (eBioscience) in RPMI 1640 medium with 10% heat-inactivated FCS (R10-FcBlock medium). In the case of Tfh staining, cells were incubated for 2 h at 37°C in R10-FcBlock with class II tetramers before a subsequent surface and viability stain in PBS.

Samples were acquired on an LSR-II flow cytometer (BD Biosciences) and analyzed using FlowJo software (Tree Star). OVA-reactive B cells were defined using fully stained cells from mice vaccinated with AdHu5-empty by setting a gate to exclude all GC B cells in these mice; as an additional negative control, the same gate was confirmed to exclude IgD^+^ B cells from AdHu5-OVA vaccinated mice. Programmed cell death protein 1 (PD1^+^) and CXCR5^+^ cells were defined using fluorescence minus one controls. OVA-MHC-II^+^ cells were defined using fully stained cells from naive mice or those vaccinated with AdHu5-empty, by setting a gate to exclude all T cells in these mice; as an additional negative control, cells were stained with a tetramer displaying the irrelevant peptide PVSKMRMATPLLMQA (human CLIP residues 87–101).

### Statistics

Prism 5 software (GraphPad) was used for the production of figures and for statistical analyses. Statistical methods used for each analysis are set out in detail in the appropriate figure legends. All cell count and Ab titer data were log_10_ transformed prior to analysis.

## Results

### Vaccine selection

Given the plethora of available platforms and dosing regimes, a comprehensive comparison of vectors and protein/adjuvant vaccination is not possible; a degree of selectivity is clearly required. Further challenges in performing any such comparison arise from uncertainties regarding the comparability of dose-response relationships between different platforms and between mice and humans. We used AdHu5-OVA at a dose of 10^8^ infectious units, MVA-OVA at a dose of 10^7^ PFU, OVA protein at a dose of 10 μg, aluminum hydroxide at a dose of 85 μg of Al^3+^ and Addavax at a dose of 25 μl; the rationale for this choice of platforms and doses is set out in detail in *Materials and Methods*. In vitro OVA expression by the AdHu5-OVA and MVA-OVA vectors was confirmed by Western blot and is shown in Supplemental Fig. 1.

### Ability of different vaccine platforms to induce primary and recall Ab responses

**FIGURE 1.**
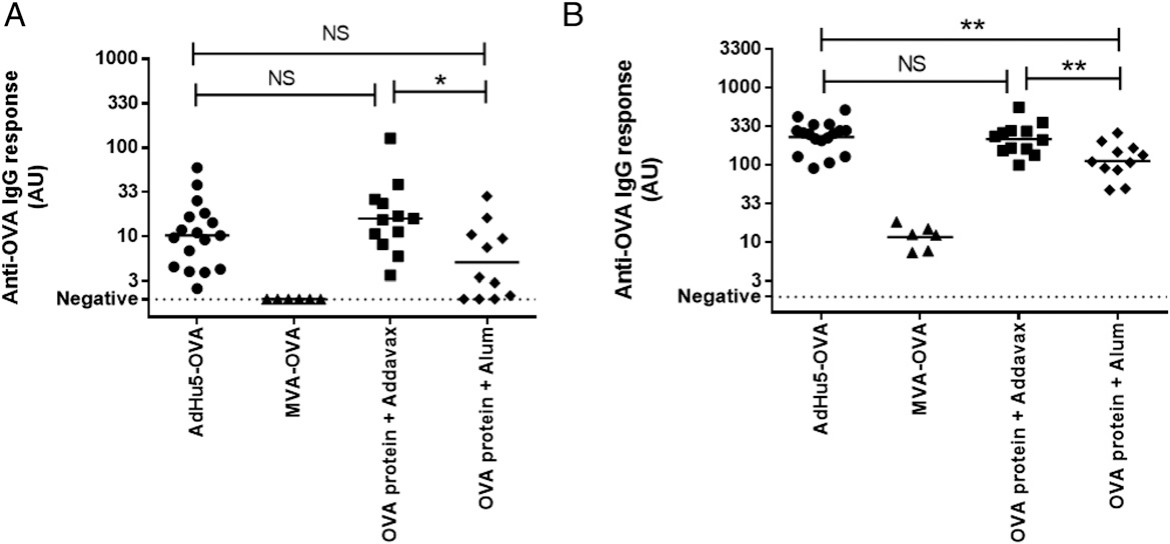
Magnitude of Ag-specific primary and recall Ab responses, C57BL/6 mice were immunized IM with AdHu5-OVA (10^8^ infectious units), MVA-OVA (10^7^ PFU), OVA protein in Addavax (10 μg in 25 μl), or OVA protein in alum at day 0 (10 μg with 85 μg of Al^3+^ as Al(OH)_3_). All animals were subsequently boosted with OVA protein in alum at week 8. IgG responses to OVA were assessed by ELISA immediately before boost vaccination (**A**) and 4 wk later [i.e., week 12 of the experiment (**B**)]. Results are expressed as AU, defined as in *Materials and Methods*. Each point represents an individual animal, with a total of 6–18 animals per condition; results shown are pooled from two identical experiments (with the exception of MVA-OVA, for which a single experiment was conducted). Lines indicate group means. Responses to AdHu5-OVA, OVA protein in Addavax, and OVA protein in alum were compared by ANOVA with Bonferroni’s posttest for pairwise comparison of individual columns: *0.01 < *p* < 0.05, **p < 0.01. NS, *p* > 0.05.

Having selected vaccine platforms and doses, we proceeded to compare the ability of the platforms to induce primary Ag-specific Ab responses (Fig. 1A), and recall responses upon re-encounter with a standardized Ag challenge, using a protein/alum boost vaccination formulation which was the same for all mice (Fig. 1B). Consistent with our previous data (8), Ag-specific Ab responses primed by AdHu5-OVA were closely comparable to those primed by OVA protein in the potent adjuvant, Addavax, with no significant difference either before or after boost vaccination with OVA in alum. Again consistent with previous experience, responses primed by AdHu5-OVA trended to be slightly higher than those induced by OVA in alum after priming and reached significantly higher levels after boosting. There was also a small but nonetheless statistically significant difference between the responses elicited by priming with OVA in Addavax versus OVA in alum, with the former eliciting higher responses both before and after boost vaccination. In contrast to the responses to AdHu5, we observed that MVA was a relatively weak priming agent, eliciting neither a strong primary Ab response nor a strong recall response upon subsequent encounter with protein Ag.

### Overall magnitude of GC responses to viral vectors is poorly correlated with humoral immunogenicity

To explore the reason for the potency of Ab induction by adenovirus (and the weak responses elicited by MVA), we sought to explore the GC responses to viral vectors as compared with protein-in-adjuvant formulations.

**FIGURE 2.**
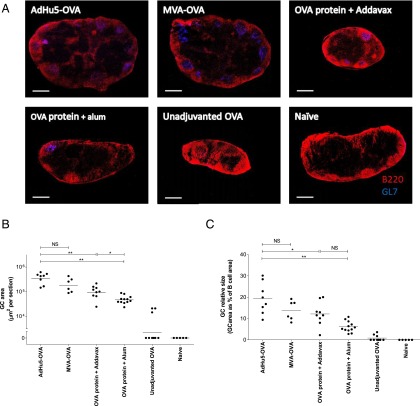
Assessment of GC responses by immunohistology. C57BL/6 mice were immunized with AdHu5-OVA, MVA-OVA, OVA protein in Addavax, OVA protein in alum, or unadjuvanted OVA protein. Immunized animals were culled at day 10 after immunization, alongside age-matched naive mice. Inguinal lymph nodes were harvested, sectioned, and stained to detect B cell zones and GC B cells. (**A**) shows representative sections from each group, with B cell zones indicated by B220 staining (red) and GC B cells indicated by GL7 staining (blue). White scale bars, 400 μm. (**B**) and (**C**) show quantitative comparison of GC area. Six to twelve individual sections were assessed for each immunization regimen (one central section from one or both inguinal lymph nodes from each of three to six mice immunized with each regimen). (B) shows the total GC area per LN section. (C) shows the proportion of the B cell area occupied by GCs in each LN section. In both (B) and (C), individual points denote individual LN sections and horizontal lines indicate group geometric means. Responses to AdHu5-OVA, MVA-OVA, OVA protein in Addavax, and OVA protein in alum were compared by ANOVA with Bonferroni’s posttest for pairwise comparison of individual columns. Selected pairwise comparisons are indicated with bars: *0.01 < *p* < 0.05, **p < 0.01. NS, *p* > 0.05.

We initially visualized the overall GC response using immunofluorescent staining of tissue sections collected at day 10 after immunization [typically close to the time of peak response to vaccination (45)]. We observed vigorous induction of GCs by both AdHu5 and MVA, with significantly weaker responses to protein-in-Addavax, whereas responses to protein in alum were slightly weaker again (Fig. 2). There was thus a discrepancy between the overall magnitude of GC responses induced and the relative ability of the different platforms to induce OVA Ag–specific primary and recall Ab responses, particularly with respect to the comparison between adenovirus and MVA vectors (Fig. 1).

We hypothesized that this discrepancy was likely to be due to a portion of the overall GC response to viral vector immunization being directed toward the vectors themselves.

### Detection of Ag-specific GC B and Tfh cell responses

**FIGURE 3.**
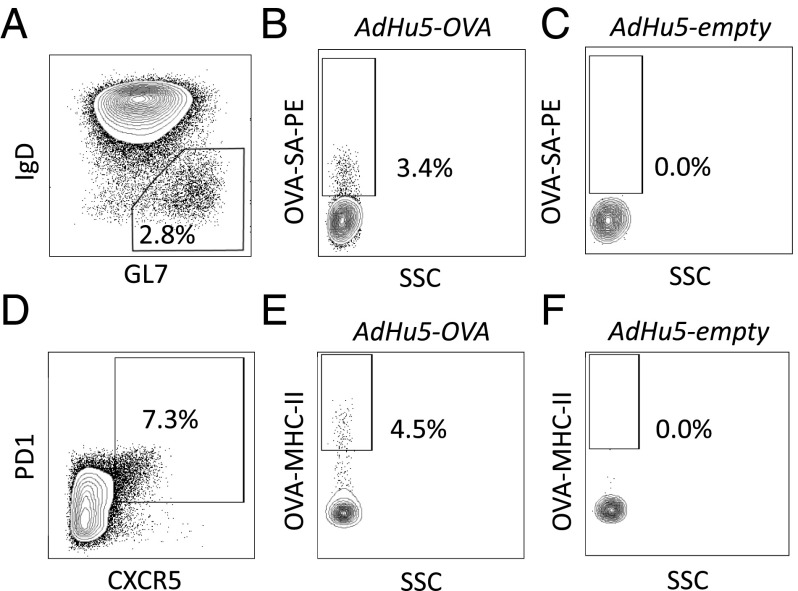
Gating for flow cytometric measurement of GC B cell and Tfh cell responses. (**A**–**C**) Representative example of GC B cell gating scheme. (A) shows gating of DLN GC B cells, defined as IgD^−^GL7^+^ after pregating on live singlet Dump^−^B220^+^ lymphocytes. OVA-SA-PE tetramer binding to GC B cells was then assessed, with gating thresholds set with reference both to IgD^+^ naive B cells within the same mouse and to GC B cells from naive mice or mice vaccinated with a non–OVA-expressing adenovirus: (B) shows OVA-SA-PE tetramer binding to GC B cells from a mouse vaccinated with AdHu5-OVA; (C) shows lack of OVA-SA-PE binding to GC B cells from a mouse vaccinated with AdHu5-empty (in addition, only 0.2% of IgD^+^ B cells in this mouse were OVA-SA-PE^+^ [data not shown]). (**D**–**F**) Representative example of Tfh gating scheme. (D) shows gating of Tfh cells, defined as CXCR5^+^PD1^+^ after pregating on live singlet CD3^+^CD4^+^CD44^+^ lymphocytes. Ag-specific Tfh cells were then identified as those binding OVA peptide–class II tetramers, with gating thresholds set with reference to Tfh cells from naive mice or mice vaccinated with a non–OVA-expressing adenovirus: (E) shows MHC-II–OVA tetramer binding to Tfh cells from a mouse vaccinated with AdHu5-OVA; (F) shows lack of MHC-II–OVA tetramer binding to Tfh cells from a naive mouse. Further controls included assessment of binding of a control tetramer [I-A(b)–human CLIP] to Tfh cells in OVA-vaccinated mice which was negligible, assessed using the same gating threshold (data not shown).

To focus on the Ag insert–specific response to vector immunization, we developed flow cytometric staining panels capable of detecting OVA-specific GC B cells and Tfh cells (Fig. 3).

After pregating on total GC B cells [defined as B220^+^ GL7^+^IgD^−^ (Fig. 3A)], we initially found that we were unable to discern Ag-specific GC B cells using either monomeric OVA labeled with a fluorophore (Life Technologies) or an in-house B cell tetramer produced by chemical biotinylation of OVA followed by incubation with streptavidin (data not shown). We subsequently developed a B cell tetramer using OVA, which had been enzymatically monobiotinylated at the C terminus. This tetramer stained a proportion of GC B cells from mice vaccinated with an OVA-expressing adenovirus but not mice which had received empty adenovirus (Fig. 3B, 3C). This site-specific biotinylation approach is similar to that used to generate MHC tetramers (46) and presumably produces a more homogeneous population of tetramers than chemical biotinylation at near-random sites. The approach is simple and readily transferable to other Ags and may therefore be of use to other investigators seeking to identify Ag-specific B cells.

There is increasing appreciation in the field of the important role of Tfh cells in orchestrating B cell responses (47). We therefore sought to quantify Ag-specific Tfh responses induced by the various platforms. We opted to use MHC-II - OVA peptide/streptavidin-fluorophore tetramers to detect OVA-specific Tfh cells in wild-type mice. These tetramers stained a proportion of Tfh cells (defined as CD44^+^PD1^+^CXCR5^+^CD4^+^ T cells) from mice vaccinated with an OVA-expressing adenovirus but not mice which had received empty adenovirus (Fig. 3D–F). This approach avoided the possibility of spurious results arising from the transfer of transgenic OVA-specific CD4^+^ T cells (OT-II), which may produce unnatural frequencies of Ag-specific T cells.

### Kinetics of responses to vaccination

Although GC responses typically peak around ten days after primary immunization with a soluble protein Ag (45), expression of Ag by adenovirus vectors does not peak until around 1 d after immunization and persists for >1 wk (12). We therefore considered it possible that the kinetics of responses to adenoviruses might be different from those of the response to soluble protein.

**FIGURE 4.**
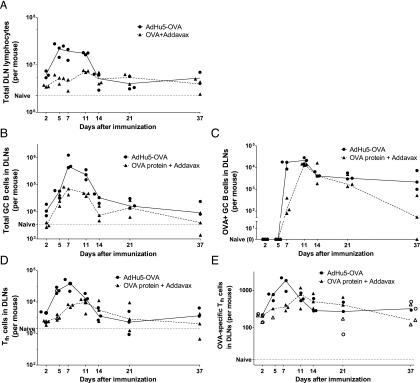
Kinetics of the GC B cell and Tfh response in mice immunized with adenoviral vectors or protein in adjuvant. DLNs were harvested at the indicated time points after immunization with AdHu5-OVA or OVA protein in Addavax; immunizations were staggered in time such that harvesting and flow cytometry was synchronous for all animals. The total number of recovered DLN lymphocytes was quantified by cell counting (**A**). Subsequently the number of GC B cells (**B**), OVA-specific GC B cells (**C**), total Tfh cells (**D**), and OVA-specific Tfh cells (**E**) were quantified by flow cytometry, using the gating strategy shown in Fig. 3. In view of the fairly substantial variation in lymph node size between animals and regimes, responses are expressed as cells in DLNs per mouse, i.e., from multiplication of the total number of lymphocytes recovered from that animal’s DLNs by the proportion of lymphocytes analyzed by flow which fell within the population of interest. Points represent individual animals (*n* = 3 per regimen per time point); lines join group means. Horizontal dotted line indicates mean number of cells in two naive animals. Open symbols in (E) represent values calculated from <10 events.

To ensure that subsequent comparisons between vector and protein-immunized animals using tissues collected at a single time point would be valid, we sought initially to compare the kinetics of responses in adenovirus and protein/Addavax immunized animals (Fig. 4).

Lymph node enlargement (reflected in elevated total DLN lymphocyte counts) was apparent between days 5 and 11 after adenovirus but not protein/adjuvant immunization (Fig. 4A). We observed that both total GC B cell and OVA Ag–specific GC B cell responses peaked between days 7 and 11 for both vector and protein/adjuvant immunized animals (Fig. 4B, 4C). Although our main focus in this study was the early period of peak GC responses, it was notable that a substantial Ag insert–specific GC B response remained detectable at day 37 after immunization with adenovirus, whereas responses to protein/adjuvant vaccination had markedly contracted by this time (Fig. 4C). Persistent GC responses may be of importance, for example, in permitting optimal memory B cell generation and somatic hypermutation.

Broadly speaking, the timing of Tfh responses was similar to that of GC B cell responses. Tfh responses did, however, expand somewhat more rapidly after immunization with adenovirus than protein/Addavax, peaking around day 7 with the former as compared with around day 11 with the latter (Fig. 4D, 4E). OVA-specific PD1^+^ Tfh cells were barely detectable by day 37 after immunization with either platform.

We (8) have previously reported Ag-specific plasma cell responses in bone marrow at late time points after vaccination with viral vectors. In this study, we briefly examined the early plasma cell responses in draining lymph nodes. Large numbers of plasma cells were present within 6 d after vaccination with adenovirus, with kinetics which differed substantially from those of GC B cells (Supplemental Fig. 2). Although it is tempting to speculate about the magnitude of this response as compared with the protein/adjuvant response, the assay used to detect these cells was not capable of discriminating between OVA-specific and adenovirus vector–specific plasma cells.

### Induction of GC B and Tfh cell responses

**FIGURE 5.**
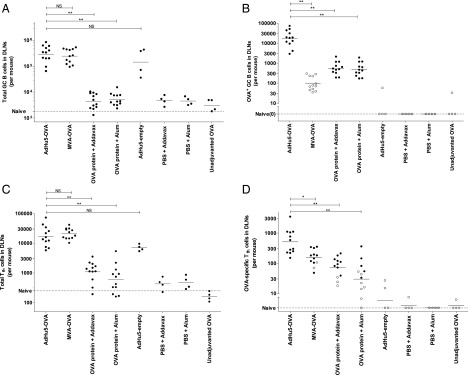
Comparison of GC B cell and Tfh cell responses to different priming vaccines. C57BL/6 mice were immunized with various regimes as indicated on graph legends. Mice were culled and DLNs harvested at day 7 after immunization, and responses assessed by flow cytometry using staining panels and gating schemes as shown in Fig. 3. (**A**) shows total GC B cell responses; (**B**) shows OVA^+^ GC B cells; (**C**) shows total Tfh responses; (**D**) shows OVA-specific (i.e., OVA-MHC-II tetramer^+^) Tfh responses. As in Fig. 3, values shown are expressed in terms of calculated number of cells in DLNs per mouse. Each point represents an individual mouse; open symbols on (B) and (D) represent figures calculated from populations comprising <10 events. Each experiment involved six mice per group with the exception of control groups (no Ag/no adjuvant) for which *n* = 2. Results are pooled from two experiments for which there was no apparent difference in cell recovery, staining intensity or intergroup pattern of responses. Horizontal lines represent geometric means for each group. Dashed horizontal lines indicate the mean size of each population in two age-matched naive mice. After log_10_ transformation of counts, responses to AdHu5-OVA, MVA-OVA, OVA protein in Addavax, OVA protein in alum [and, in the case of (A) and (C), AdHu5-empty] were compared by ANOVA with Bonferroni’s posttest for pairwise comparison of individual columns. Selected pairwise comparisons are indicated with bars: *0.01 < *p* < 0.05, **p < 0.01. NS, *p* > 0.05.

Having defined the kinetics of the responses, we proceeded to use our flow cytometry assays to compare in more detail the magnitude of GC B and Tfh cell responses induced by the different vaccine platforms around the peak time point (Fig. 5); an assumption was made that the peak time point for responses after MVA and protein/alum immunization would be similar to that after adenovirus or protein/Addavax immunization. The profile of overall GC B cell and Tfh induction was, as expected, largely parallel to the size of GCs visualized by immunofluorescence after immunization with each platform (Fig. 4). Whereas only marginal total GC B cell responses were apparent when Addavax or alum were given without Ag, the magnitude of the response to an adenovirus lacking an inserted Ag transgene was closely similar to that induced by the OVA-expressing adenovirus (Fig. 5A).

A different pattern was apparent when examining Ag insert–specific B cells. Although adenovirus resulted in greater populations of both total GC B cells and OVA-specific GC B cells than in protein–adjuvant vaccination, MVA induced very few OVA-specific GC B cells despite its ability to induce a vigorous overall response (Fig. 5B). The very high ratio of non-insert–specific to Ag insert-specific GC B cells after MVA immunization may reflect the antigenic complexity of the large MVA virion.

Total Tfh responses to the viral vector platforms were greater than those to the protein-in-adjuvant vaccines (Fig. 5C). For all platforms, the proportion of total Tfh cells, which detectably bound MHC-II tetramers was typically <5%; nonetheless, Ag-specific Tfh responses were clearly detectable above background levels. OVA-specific Tfh responses were significantly higher in adenovirus-vaccinated mice than in other groups (Fig. 5D). This difference was no longer apparent at day 10, consistent with findings from our kinetic analysis suggesting that at this time point, when the Tfh response in protein/adjuvant immunized mice reached its peak, the response in adenovirus-immunized mice had begun to decline (data not shown). OVA-specific Tfh responses to MVA compared favorably to those induced by the protein-in-adjuvant formulations, suggesting that the poor Ag-specific GC B cell and IgG responses elicited by the MVA vector were probably not attributable to limited Tfh frequency.

### Non–transgene-expressing adenovirus does not adjuvant responses to protein

The ability of adenovirus to induce robust adaptive immune responses appears to be dependent on innate immune activation (14–17). It is unclear whether the Ag needs to be expressed in the same cells experiencing virus-triggered innate activation (Ag in cis), or whether the degree of spatiotemporal linkage between the protein Ag and the virus which is necessary might be sufficiently loose to permit a non-transgene-expressing empty adenovirus to adjuvant the immunogenicity of a coadministered recombinant protein (Ag in *trans*).

**FIGURE 6.**
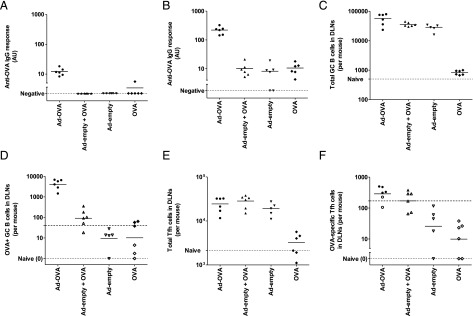
Lack of adjuvantation of responses to protein by empty adenovirus. Mice were immunized with each of the indicated regimes at day 0. A first cohort of mice was boosted with OVA in alum at day 54; a second cohort of mice was culled and DLNs harvested at day 12. (**A** and **B**) depict IgG responses in the first cohort at day 54 (preboost) and day 85 (postboost), respectively. Results are expressed in AU (see *Materials and Methods*). (**C**–**F**) depict responses in DLNs of the second cohort at day 12: total GC B cell response (C); OVA-specific GC B cell response (D); total Tfh response (E); and OVA-specific Tfh response (F). Gates for Ag-specific cells (D and F) were set with respect to naive mice. In each panel, points represent individual mice (*n* = 5–6 per group) with open points in (D) and (F) representing values calculated from <10 events. Lines represent group geometric means. The labeled horizontal dotted line represents the mean response observed in two naive mice. To aid interpretation of the weak responses observed in the non–Ad-OVA groups, the upper dashed horizontal line in (D and F) provide an indication of background (the mean plus two standard deviations of the “response” in the Ad-empty group).

Fig. 6 shows that, in fact, there was little or no effect of coadministered empty adenovirus upon Ab or cellular responses. There was no statistically significant difference between IgG responses to unadjuvanted OVA protein and OVA protein mixed with empty adenovirus at preboost or postboost timepoints (Fig. 6A, 6B, *p* > 0.05 by two-tailed *t* test). Total GC B cell and total Tfh cell responses to the mixture of OVA protein with empty adenovirus were comparable to the responses to empty adenovirus alone (Fig. 6C, 6E). OVA-specific GC B cell and Tfh cell responses to the protein/empty adenovirus mixture were barely detectable (Fig. 6D, 6F) and, particularly in the case of the OVA-specific GC B cell response, considerably lower than the response to AdHu5-OVA.

## Discussion

Comparison of the properties of very different vaccine platforms can be a challenging task but is necessary to guide the development and prioritization of novel approaches. There are few Ag–platform combinations for which such assessments have been undertaken in humans. Preclinical assessments carry the benefit of access to tissues, which cannot be reached in humans, at the expense of a number of drawbacks including uncertainty regarding the comparability of animal dosing to safe and tolerable human dosing.

In this study, we sought to compare humoral responses to two viral vector and two protein/adjuvant vaccine platforms in mice, using a full-length, structured protein Ag (as opposed to a hapten) and the clinically relevant i.m. route of immunization. We focused our assessment on IgG responses and the GC response to primary immunization and chose to use assays capable of detecting Ag-specific GC B cell and Tfh cell responses in wild-type mice in preference to an artificial system using transgenic T cells. Given that the rarity of MHC-II tetramer-binding Tfh cells made it challenging to quantify responses in some experiments, it would clearly be of interest to replicate our experiments using adoptively transferred transgenic T cells. Nonetheless, clear responses were detectable under most conditions using our assays.

There is currently a contrast between increasing interest in the development of adenovirus vectors for Ab induction (notably in the response to the recent Ebola epidemic) and a persistent belief in some quarters that viral vectors are second best options for Ab induction, inferior to protein/adjuvant vaccines. Our current data provide further support for the notion that adenovirus vectors are potent inducers of B cell responses, inducing not only IgG responses but also GC B cell and Tfh cell responses, which compare favorably to those induced by leading clinically relevant protein/adjuvant formulations. Our observation of potent Ag-specific GC B cell responses to adenoviruses goes some way toward explaining our previous data, suggesting that priming immunization with a recombinant adenovirus can overcome the requirement for the inclusion of a potent adjuvant with a protein boost to reach optimal postboost Ab titers: the robust GC responses to adenovirus-delivered transgene are likely to result in a substantially expanded Ag-specific memory B cell pool. The magnitude of adenovirus-induced memory B cell responses and the extent of somatic hypermutation—both of which are largely GC-dependent processes—will clearly be worthwhile topics for future investigation.

Our data underscore the importance of assessing Ag-specific rather than total GC responses when comparing viral vectors with each other and with protein/adjuvant formulations. Unsurprisingly, both adenovirus and MVA (at doses of 10^8^ infectious units and 10^7^ PFU, respectively) induced larger populations of non–insert-specific GC B cells than protein/adjuvant vaccination. In the case of MVA, the non–insert-specific response was dominant to the extent that only a marginal GC B cell response was detectable to the inserted vaccine Ag: possible explanations for this might include a high ratio of viral:transgene protein expression, the transience of Ag expression after MVA immunization, and the induction of dendritic cell apoptosis by MVA (12, 48). Although it has been observed that the ability of vaccinia to achieve Ab-mediated protection against smallpox is encouraging for the use of poxvirus vectored vaccines for Ab induction (6), caution is clearly required regarding their ability to induce responses to soluble secreted inserted Ags in Ag-naive animals. The situation in Ag-experienced animals, in which the available Ag-specific B cell pool will be larger, is likely to be different: in this context, the Ag-specific B cell response to poxvirus vaccination may well constitute a considerably greater proportion of the total response.

Viral vectors are better known for their ability to induce T cell responses than their induction of Ab. We and others have previously speculated that induction of Tfh cells by viral vectors could be superior to that by protein/adjuvant formulations, and that this could partly explain the robust ability of adenoviruses to prime humoral responses.

Overall, the magnitude of Tfh responses measured in this study correlated fairly poorly with Ab immunogenicity as measured by ELISA. Although OVA-specific Tfh responses at day 7 after vaccination with adenovirus were significantly higher than those induced by other platforms (consistent with the good Ab induction by adenovirus), Tfh responses to MVA trended to be higher than those to protein-in-adjuvant formulations, yet the humoral immunogenicity of MVA was extremely poor. There is clearly scope for further study in this area: notably, comparative analysis of the functional attributes of the Tfh responses elicited by the different regimes would be of interest, as they may differ in the Th1/Th2 bias of the T cell responses they elicit, resulting in a qualitatively different Tfh response (47).

Relationships between measured responses in DLNs and humoral responses were imperfect: Ab induction by Ad-OVA was similar to that by protein/Addavax, despite superior OVA-specific GC B and Tfh responses; conversely, Ab induction by protein/Addavax was superior to protein/alum, despite similar levels of Ag-specific GC B and Tfh cells. It seems likely that, under these conditions, once a certain level of GC B and Tfh response is reached, some additional unmeasured factor becomes limiting upon the IgG response.

Finally, our results suggest that there is little effect of non–insert-expressing adenovirus upon the humoral response to a coadministered protein Ag. Spatiotemporal association of adjuvant-induced innate immune signaling and Ag availability has been shown to be critical for the immunogenicity of other vaccine platforms (49). Although our results do not prove that the Ag needs to be expressed from the same cell which is infected by the adenovirus vector, they suggest that the spatiotemporal linkage between coadministered protein and adenovirus is insufficiently tight to achieve adjuvanticity.

Overall, our results provide further encouragement for the use of adenoviral-vectored vaccines for Ab induction. Our clinical trial data and that of others demonstrate that this platform is capable of robust humoral immunogenicity in humans (25, 50, 51). Ultimately, the most appropriate choice of vaccine platform for a given disease target is becoming increasingly nuanced. For diseases which require the induction of Ab responses in Ag-naive subjects against proteins, which are difficult to produce with conformational accuracy in vitro—notably many viral glycoproteins and some eukaryotic parasite Ags—adenoviruses are an increasingly attractive choice.

## Supplementary Material

Data Supplement
