# Supplementary material for: Germinal Center B Cell and T Follicular Helper Cell Responses to Viral Vector and Protein-in-Adjuvant Vaccines
Source: J Immunol. 2016 Jul 13;197(4):1242–51. doi: 10.4049/jimmunol.1502472 (PMC4974488; doi:10.4049/jimmunol.1502472)
Supplement: Data Supplement [file JI_1502472.zip › JI_1502472_Supplemental_Material_1.pdf]

Supplementary Information

Supplementary Table 1: Staining reagents

Abbreviations used in 'Assay' column: IH- immunohistology; BCF- B cell flow cytometry; TCF- T_{fh} cell flow cytometry.

Assay	Antigen/ target	Clone/ Reagent	Fluorochrome/ Label	Supplier	Catalogue number	Dilution
IH	GL7	GL7	Pacific Blue	eBioscience	144613	100
IH	B220	RA3-6B2	Biotin	eBioscience	13-0452-85	500
IH	N/A	Streptavidin	Alexa 594	Life Technologies	A-11005	1000
BCF	CD138	281-2	Brilliant Violet 421	BioLegend	142508	50
BCF	IgD	11-26c	PerCP-eFluor 710	eBioscience	46-5993-80	1000
BCF	Viability	Live/Dead	Fixable Red	Life Technologies	L-23102	1000
BCF	IgG1	RMG1	Alexa 488	BioLegend	406605	400
BCF	IgG2a	RMG2a	Alexa 488	BioLegend	407105	400
BCF	IgG2b	RMG2b1	Alexa 488	BioLegend	406705	400
BCF	OVA-BCR	B cell tetramer	Phycoerythrin	Produced in-house	N/A	2.5 µg/mL
BCF	CD4	RM4-5	APC-Alexa 780	eBioscience	47-0042-82	150
BCF	CD8	53-6.7	APC-Alexa 780	eBioscience	47-0081-82	150
BCF	F4/80	BM8	APC-Alexa 780	eBioscience	47-4801-82	150
BCF	CD11c	N418	APC-Alexa 780	eBioscience	47-0114-82	150
BCF	Gr1/Ly6G	RB6-8C5	APC-Alexa 780	eBioscience	47-5931-82	150
BCF	B220	RA3-6B2	Alexa 700	eBioscience	56-0452-80	50
BCF	GL7	GL7	eFluor 660	eBioscience	50-5902-82	50
TCF	CD4	RM4-5	qDot 605	Life Technologies	Q10092	1350
TCF	Live/Dead	N/A	Aqua	Life Technologies	L34957	1000
TCF	OVA-TCRs	See text	Phycoerythrin	NIH Tetramer Core Facility	22629, 22630, 22631	50
TCF	PD1	J43	PerCP-e710	eBioscience	46-9985-82	450
TCF	CXCR5	2G8	PECy7	BD Pharmingen	560617	50
TCF	CD3	17A2	APC-Cy7	Biolegend	100222	150
TCF	CD44	IM7	Alexa 700	eBioscience	56-0441-82	150

Supplementary Figure 1

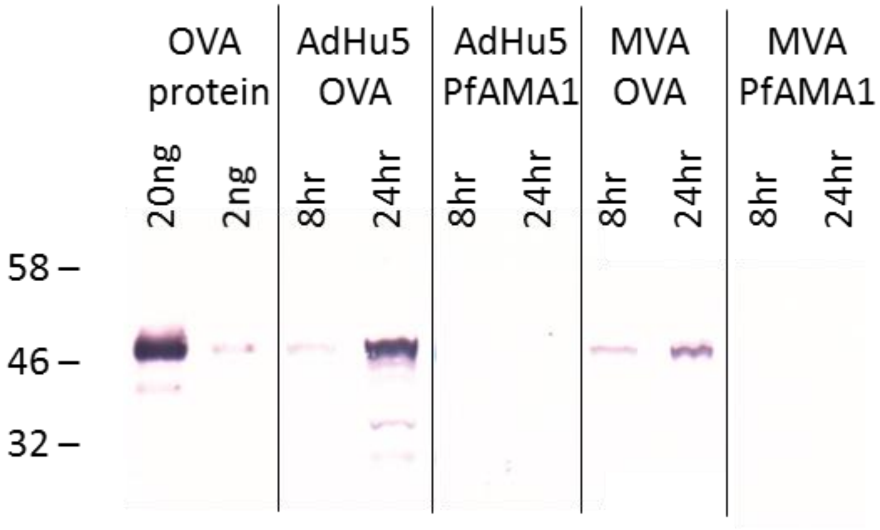

Supplementary Figure 1: Expression of ovalbumin by vectors
Ovalbumin was detected by Western blotting using supernatant from culture of vector infected cells, as described in Methods. Recombinant ovalbumin (20 ng and 2 ng in adjacent lanes) was used as a positive control. Positions of molecular weight standards (kilodaltons) are shown to the left of the image. Very similar results were obtained using infected cell lysate (data not shown).

Supplementary Figure 2

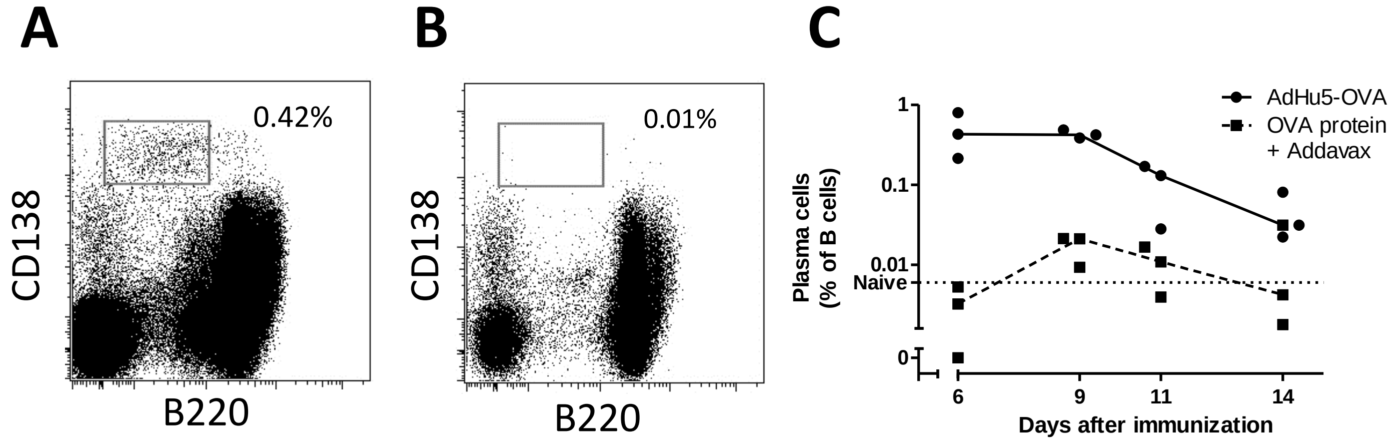

Supplementary Figure 2: Kinetics of plasma cell responses in lymph nodes
Panels A and B show gating of plasma cells, defined as CD138^{hi} B220^{int} after pre-gating on live singlet Dump^{-} lymphocytes, at day 9 after immunization with AdHu5 OVA (A) and in a non-immunized animal (B).

Panel C: DLNs were harvested at the indicated timepoints after immunization with AdHu5-OVA or OVA protein in Addavax. Plasma cells were enumerated by flow cytometry, using the gating strategy shown in panels A and B. Points represent individual animals (n=3 per regime per timepoint); lines join group medians. Horizontal dotted line indicates median number of cells in two naïve animals.
